# PGPR and nutrient consortia promoted cotton growth, antioxidant enzymes, and mineral uptake by suppressing sooty mold in arid climate

**DOI:** 10.3389/fmicb.2025.1551465

**Published:** 2025-03-20

**Authors:** Muhammad Luqman, Maqshoof Ahmad, Abubakar Dar, Azhar Hussain, Usman Zulfiqar, Muhammad Zahid Mumtaz, Adnan Mustafa, Abd El-Zaher M. A. Mustafa, Mohamed S. Elshikh

**Affiliations:** ^1^Department of Soil Science, The Islamia University of Bahawalpur, Bahawalpur, Pakistan; ^2^Department of Agronomy, Faculty of Agriculture and Environment, The Islamia University of Bahawalpur, Bahawalpur, Pakistan; ^3^Institute of Molecular Biology and Biotechnology, The University of Lahore, Lahore, Pakistan; ^4^College of Agronomy, Gansu Agricultural University, Lanzhou, China; ^5^South China Botanical Garden, Chinese Academy of Sciences, Guangzhou, China; ^6^Department of Botany and Microbiology, College of Science, King Saud University, Riyadh, Saudi Arabia

**Keywords:** sooty mold, balanced nutrition, PGPR, nutrient uptake, sustainable agriculture

## Abstract

**Introduction:**

Cotton (*Gossypium hirsutum* L.) plays a vital role in Pakistan’s economy, providing significant employment opportunities and supporting the country’s textile industry. However, cotton productivity is severely impacted by pests and diseases, such as black spots caused by sooty mold, posing critical challenges to sustainable agriculture. This study investigates a novel integration of plant growth-promoting rhizobacteria (PGPR) with recommended NPK fertilizers and micronutrients to enhance cotton growth, yield, disease resistance, and post-harvest soil properties.

**Methodology:**

A consortium of *Bacillus megaterium* (ZR19), *Paenibacillus polymyxa* (IA7), and *Bacillus* sp. (IA16) were evaluated under six treatments: control (T1), PGPR (T2), recommended NPK (T3), recommended NPK + PGPR (T4), recommended NPK + micronutrients (T5), and recommended NPK + micronutrients + PGPR (T6).

**Results:**

The results depicted a significant increase in antioxidant activities of 19% in superoxide dismutase (SOD), 29% peroxidase (POX), 28% peroxidase dismutase (POD), and 14% catalase (CAT) activity under T6 as compared to control. Similarly, growth parameters substantially improved root length (39%), shoot length (19%), and root and shoot biomass by up to 31 and 20%, respectively, under T6. Moreover, the yield attributes like single boll weight and lint percentage were also enhanced by 32 and 13%, respectively, under the integration. In contrast, the PGPR consortium demonstrated considerable biocontrol potential against sooty mold, as disease incidence was reduced by 68% in cotton, the disease index was 75%, and control efficacy reached 75%. The PGPR consortium also substantially improved post-harvest soil biological and chemical properties, including bacterial populations, microbial biomass nitrogen, organic matter, and essential nutrient availability.

**Discussion:**

So, these findings witnessed the dual behavior of the *Bacillus* and *Paenibacillus* strains with balanced nutrition and can lead us to the development of an effective biopesticide cum biofertilizer for the sustainable production of cotton in arid conditions by combating sooty mold effectively.

## 1 Introduction

Cotton is the backbone of Pakistan’s economy, playing a vital role in providing employment opportunities and supplying raw materials for the textile industry (Rana et al., 2020). Globally, Pakistan is the 4th largest cotton producer and ranks as the 3rd largest consumer of cotton. The textile sector is the country’s largest industrial domain, employing approximately 40% of the workforce (Economic Survey of Pakistan, 2022; Mehmood et al., 2021; GOP, 2019). Beyond its significance as a fiber crop, cotton is also a key oilseed crop in Pakistan, alongside other major oilseeds like sunflower, canola, and rapeseed (PBS, 2022).

Cotton is grown mainly in arid and semi-arid regions due to its lower water requirements than other cash crops (Chen et al., 2019). However, high temperatures and drought stress in these areas exacerbate sooty mold severity, as plants under stress are more susceptible to pest and fungal attacks. These regions are less fertile and have poor nutrient availability, especially having low diffusion coefficients (Zhang et al., 2017). Agricultural productivity in these regions mainly depends upon the agrochemicals (fertilizers, pesticides) as an integral part of farming systems in these regions. Applying fertilizers and enhancing crop production are responsible for enhancing input costs and environmental deterioration (Ali et al., 2023). For example, despite the abundance of phosphorus in soils, it often remains unavailable and insoluble for plants, making it a major limiting factor in crop production and may accumulate in surface water by soil erosion and runoff from the fertilized field (Mahmood et al., 2024).

Another alarming threat in the arid regions is the attack of sooty mold, which has become a significant threat to cotton production worldwide in the last decade (Belachew and Jenber, 2024). Sooty mold (black fungus) thrives on honeydew secretions from insect pests, *viz*. aphids and whiteflies, forming a black, soot-like coating on cotton leaves and bolls, impairing photosynthesis and reducing crop yield by 40% under higher infestation (Mondal et al., 2020). *Bemisia tabaci* is one of the most destructive pests among 160 insect pests of cotton throughout its growth (Kouser et al., 2019; Naeem-Ullah et al., 2020). *B. tabaci* not only causes direct damage but also facilitates the growth of black fungus through its gummy secretions, impairs photosynthetic activity, and can lead to plant death (Shah et al., 2020). Along with yield reduction, sooty mold deteriorates the fiber strength and lint quality, leading to price penalties for affected cotton (Wrather et al., 2008). Chemical fungicides are used to control the black fungus infection in cotton, but none has been found effective and registered against sooty mold control (Hameed et al., 2023). Moreover, the increased dosage and repeated use may cause fungicide resistance in the fungus and deteriorate the environmental quality (Ziółkowska et al., 2021; Tooker and Pearsons, 2021). Approximately 0.3 billion US$ has been spent annually on pest control, of which 80% is used on cotton crops alone (Khan et al., 2015; Shuban et al., 2024).

Growing concerns about human and environmental health have prompted researchers to shift their focus from synthetic products to safer alternatives for enhancing nutrient use efficiencies and pathogen control (Lahlali et al., 2022). Numerous studies have urged the use of sustainable options for safe crop production and controlling crop pests (Abdullah and Zahoor, 2023; Ayilara et al., 2023; Hezakiel et al., 2024; Dar et al., 2024a, 2024b). One such strategy is to use plant growth-promoting rhizobacteria (PGPR), which reside in the rhizosphere and compete with other microorganisms for food and survival (Kloepper and Okon, 1994). The PGPRs increase crop production following various direct and indirect mechanisms. The direct mechanisms involved nitrogen fixation, phytohormones production [gibberellins, auxins (IAA) and cytokinins], nutrient solubilization (phosphorus, potassium, iron, and zinc), siderophores and exopolysaccharides production (Islam et al., 2013: Maheshwari et al., 2015; Murad et al., 2024). Whereas PGPRs indirectly boost crop growth, *viz.* antibiotics production, lytic enzymes, ACC-deaminase, hydrogen cyanide (HCN), competition, induction of systemic resistance, and secondary metabolites production to cope with crop pests (pathogens and weeds), and abiotic stress tolerance (Beneduzi et al., 2012; Ramadan et al., 2016; Ajinde et al., 2024; Dar et al., 2024a). These bacteria increase nutrient concentration by nutrient solubilization in soil, improving nutrient availability and plant uptake (Hussain et al., 2019). PGPRs alleviate stress and enable crop production under abiotic stress, *viz.* drought (Arzanesh et al., 2011), salinity (Arora et al., 2012), and flooding stress (Tewari and Arora, 2016). In addition, these are also the major contributors to the bioremediation of metal-polluted sites (Zheng et al., 2024; Guo et al., 2021; Kong and Glick, 2017; Mishra et al., 2017).

With the advancement of research and development, the indirect mechanisms of PGPR are being used to suppress pests (weeds and pathogens) in field crops (Hassan et al., 2024). Recently, bacteria, one of the safe alternatives to pesticides, has shown promising results in alleviating sooty mold damage (McLaughlin et al., 2023). Certain strains of *Bacillus subtilis* and *Pseudomonas fluorescens* have demonstrated their antifungal properties by producing secondary metabolites against sooty mold and reducing disease severity by up to 60% in field trials (Kumar et al., 2017). Singh et al. (2016) reported the induction of systemic resistance to boost defense mechanisms and antifungal activities in cotton crops.

The knowledge gap lies in integrating PGPR and balanced nutrition to control cotton sooty mold. Therefore, the present study was designed to test the dual action of PGPR and balanced nutrition for plant growth promotion and fungal disease suppression for sustainable cotton production. This study aimed to investigate the synergistic impact of a specific PGPR consortium (*Bacillus megaterium* ZR19, *Paenibacillus polymyxa* IA7, and *Bacillus* sp. IA16) in combination with balanced nutrition (NPK and micronutrients) application on the growth of two native cotton varieties of Pakistan (IUB13 and IUB4). We also aimed to integrate the PGPR consortium and balanced nutrition to promote nutrient uptake and control sooty mold attacks on cotton. Thus, the novelty of this study lies in using bacteria with dual functions of growth promotion and sooty mold suppression with balanced nutrition, which provides valuable insights for sustainable cotton production tailored to the challenging arid climate. Previously, we explored the PGPR and balanced fertilizers individually for cotton growth (Unpublished). However, there is a gap in integrating PGPR and balanced nutrition for sooty mold control, which we have explored in the present investigation.

## 2 Materials and methods

### 2.1 Collection of rhizobacterial strains and cotton seeds

Three rhizobacterial strains were obtained from the culture bank of Soil Microbiology and Biotechnology Laboratory, Department of Soil Science, The Islamia University of Bahawalpur. These bacterial strains were identified as *Bacillus megaterium* ZR19 (MN007186) by Iqbal et al. (2020) and *Paenibacillus polymyxa* IA7 (NM005923) and *Bacillus* sp. IA16 (NM005924) by Ahmad et al. (2021). These strains could solubilize insoluble minerals and demonstrated the production of siderophores, exopolysaccharides, and ammonia, and were positive for cellulase and protease activities (Iqbal et al., 2020; Ahmad et al., 2021). Moreover, these bacterial strains were compatible with growing simultaneously. Seeds of two cotton varieties, i.e., IUB13 and IUB4, were collected from the National Cotton Breeding Institute (NCBI), The Islamia University of Bahawalpur. These cotton varieties were selected for this study due to their local farmer preference and possess high yield potential in the study area, making them relevant for regional agricultural practices.

### 2.2 Preparation of inoculum and seed inoculation

The collected strains were grown in DF (Dworkin and Foster) salt minimal media (Mumtaz et al., 2022) for 48 h at 100 rpm shaking and 28 ± 2°C to inoculate the cotton seeds. Before coating, seeds were surface sterilized by using ethanol (95%) and HgCl_2_ (0.2%) and then washed gently with sterilized water (Abd-Alla et al., 2012). The bacterial consortium was developed in a sterilized media storage bottle by taking equal volumes of three bacterial strains in a 1:1:1 ratio of the bacterial cultures and homogenized through vortexing. The cotton seeds of both varieties were coated with the bacterial culture by a slurry-based carrier coating prepared by mixing the inoculum with sterile peat and sugar solution in a 4:5:1 ratio, as reported in our previous work (Mumtaz et al., 2022).

### 2.3 Pot trial

The effectiveness of bacterial strains was tested along with chemical fertilizers to boost cotton growth and yield under natural conditions. The treatments *viz.* treatments: control (T1), PGPR (T2), recommended NPK (T3), recommended NPK + PGPR (T4), recommended NPK + micronutrients (T5), and recommended NPK + micronutrients + PGPR (T6) were laid out on a completely randomized design (CRD) under factorial settings. Six cotton seeds were sown in each earthen pot with dimensions of 18˝ × 12˝ (height × diameter) filled with 10 kg sieved (using 2 mm mesh) and dried soil. The soil was obtained from a farmer’s field and characterized for the physicochemical attributes (Table 1) using the methods detailed in Handbook 60 (Regional Salinity Laboratory Staff (US), 1954). Recommended fertilizer [urea was used as the source of nitrogen (N), diammonium phosphate for nitrogen (N) and phosphorus (P), and muriate of potash for potassium (K) doses (NPK at 310:170:110 kg ha^−1^, respectively)] were applied in the pots as basal doses. Nitrogen fertilizer was applied in 3 equal split intervals, including basal, early flowering, and early bol formation stages. These fertilizer sources were chosen based on their availability, effectiveness, and cost-efficiency for the cotton crop, ensuring optimal nutrient supply. The trial was conducted in the wirehouse of the Department of Soil Science, The Islamia University of Bahawalpur. The pots were regularly irrigated with good-quality water to fulfill the irrigation requirements. Antioxidant enzymatic status was estimated at the flowering stage and other growth and yield parameters were determined during harvesting. The biocontrol potential of the strains was evaluated by spraying the PGPR consortium (2 liters per hectare in 1:1:1 ratio for each strain) in T2, T4, and T6 treatments at 55, 85, and 115 days after germination. While T3 and T5 were sprayed with pyriproxyfen (Axxiprox, Swat Agro Chemicals) at 55 days, acephate (FMC) at 85 days, and acetamiprid (Mospilan, Arysta Life Sciences) at 115 days after cotton germination.

**Table 1:** Soil pre-sowing analysis for physicochemical attributes.

Characteristics	Value
pH_s_	7.8
EC_e_	1.64 dS m^−1^
Sand	45%
Silt	42%
Clay	13%
Textural class	Loam
Saturation percentage	36%
Total nitrogen	0.022%
Available phosphorous	5.2 mg kg^−1^
Extractable potassium	80 mg kg^−1^
Organic matter	0.29%
Iron	3.8 mg kg^−1^
Zinc	0.66 mg kg^−1^

### 2.4 Growth and yield properties

The growth parameters, such as root and shoot lengths, were measured using a meter rod, and the root and shoot fresh using a portable balance immediately after harvesting. The cotton bolls were counted manually, and the seed cotton from the open boll was picked manually and weighed through a portable weight balance. Lint yield was determined by separating the cotton and seeds.

### 2.5 Biocontrol efficacy of *Bacillus megaterium* (ZR19), *Paenibacillus polymyxa* (IA7) and *Bacillus* sp. (IA16)

The efficacy of the *Bacillus megaterium* (ZR19), *Paenibacillus polymyxa* (IA7), and *Bacillus* sp. (IA16) consortium as a biocontrol agent against sooty mold was evaluated through a foliar application (spray) on cotton plants, and the effect was compared to the impact of synthetic chemicals spray. Disease severity was assessed using a standard rating scale from 0 to 4, quantifying the percentage of plant tissue exhibiting symptoms such as chlorosis, leaf necrosis, or defoliation (0 = healthy plant, 1 = 1–33% affected, 2 = 34–66% affected, 3 = 67–99% affected, 4 = dead plant). Disease incidence (percentage of infected plants), disease index (average disease severity), and control efficacy (relative reduction in disease incidence) were calculated from the Equations 1–3 developed by Zhu et al. (2013) given below:


(1) $$\mathrm{Disease}\;\mathrm{incidence}\;\left(\%\right)=\left[\left(n_{1}+n_{2}+n_{3}+n_{4}\right)/n\right]\times 100$$

(2) $$\mathrm{Disease}\;\mathrm{index}=\left[\left(0n_{0}+1n_{1}+2n_{2}+3n_{3}+4n_{4}\right)/4n\right]\times 100$$

(3) $$\mathrm{Control}\;\mathrm{efficacy}\;\left(\%\right)=\left[\frac{\left(\begin{array}{l} \mathrm{Disease}\;\mathrm{inde}x_{\mathrm{control}}-\\ \mathrm{Disease}\;\mathrm{inde}x_{\mathrm{treatment}} \end{array}\right)}{\mathrm{Disease}\;\mathrm{inde}x_{\mathrm{control}}}\right]\times 100$$

Where *n*_0_-*n*_4_ represents the number of plants assigned to each corresponding disease rating, and *n* denotes the total number of plants assessed.

### 2.6 Determination of antioxidant enzymatic status

The fresh leaf sample of 0.25 g was thoroughly mixed with 4 mL of pre-cooled phosphate buffer solution (pH 7.8; 0.0663 g of NaH_2_PO_4_.2H_2_O + 16.385 g of Na_2_HPO.12H_2_O dissolved into 1,000 mL distilled water) in a pre-cooled mortar placed on ice. The homogenized mixture was centrifuged for 20 min at 4°C and 10,000 rpm to collect enzyme extract. After that, the supernatant was taken in Eppendorf tubes and analyzed for antioxidant enzyme activities. For ascorbate peroxide, 2.5 mL of phosphate buffer solution was used, and reading was noted on a spectrophotometer at 290 nm wavelength by following the method of Prochazkova et al. (2001). Catalase and peroxidase activity was measured by following the protocol of Chance and Maehly (1955) at 240 and 470 nm wavelengths, respectively. The assay mixture was prepared by mixing 2.6 mL of 1 mM KH₂PO₄ buffer, 400 μL of H₂O₂, and 40 μL of enzyme extract. The process explained by Giannopolitis and Ries (1977) was employed to assess the superoxide dismutase activity at 560 nm. Antioxidant activities were expressed in terms of units per mg fresh leaf weight (U mg^−1^ FW).

### 2.7 Macro and micronutrients determination in roots and shoots

Wet digestion of plant samples was done by following the protocol described by Wolf (1982). Five mL of the digested sample was added to the Kjeldahl flask and attached to the Kjeldahl distillation unit by adding 10 mL NaOH (40%). A conical flask containing 5 mL of boric acid (4%) was attached at the receiving point. After collecting about 30–40 mL distillate, the flask was removed from the distillation unit and 5–10 drops of mixed indicator were added. The flask contents were titrated against 0.01 N standard sulfuric acid solution until the pink endpoint. Phosphorus was determined in digested plant samples by adding Barton reagent (Ashraf et al., 1992). For this purpose, 5 mL of aliquot was taken in a 50 mL volumetric flask and 10 mL of Barton reagent. After incubating for 30 min the readings were measured on a UV–visible spectrophotometer at 420 nm (Model G6860A, Agilent Technologies, Australia) and compared with the standard curve of known concentration potassium dihydrogen phosphate standards. Potassium concentration was determined from the digested samples by using a flame photometer (Model: BWB-XP, BWP Technologies, United Kingdom). The concentration of K was calculated by comparing instrument readings with the KCl calibration curve. The Fe and Zn concentrations in samples were determined using an Atomic Absorption Spectrophotometer (Model 240FS AA, Agilent Technologies Australia).

### 2.8 Post-harvest soil sample collection and analysis

After crop harvest, post-harvest rhizosphere soil samples were collected, air-dried, and sieved through a 2-mm sieve and stored at 4°C. The prepared soil samples were analyzed within 5 days for biological properties, including bacterial population, microbial biomass N, and organic matter. The bacterial population was enumerated in terms of colony-forming units (cfu) using standard serial dilution and pour plate technique (Alexander, 1982). Microbial biomass nitrogen was calculated by subtracting the biomass nitrogen in chloroform-fumigated soil from the non-fumigated soil sample using the method developed by Okalebo et al. (1993). For the analysis of organic matter, the method of Nelson and Sommers (1982) was used. To ensure data reliability and precision, all measurements were conducted in triplicates. The soil’s chemical properties, including ammonium N, nitrate N, available phosphorus, and extractable potassium, were also analyzed using standard protocols. Ammoniacal N and nitrate N were determined using the methods of Kamphake et al. (1967) and Sims and Jackson (1971), respectively. The available phosphorus was determined according to Watanabe and Olsen’s (1965) method, and the extractable potassium was noted using a flame photometer (Model: BWB-XP, BWP Technologies, United Kingdom).

### 2.9 Statistical analysis

The obtained data was statistically analyzed by performing a two-way ANOVA interaction at Statistix 8.1 Analytical Software, Tallahassee, Florida (Steel et al., 1997). Treatment means were computed using an honestly significant difference test (HSD) at 5% probability. The graphs were prepared using R studio. The principal component analysis (PCA) and Pearson’s correlation among growth, biochemical, antioxidants, yield attributes of cotton, and biocontrol potential of treatments was performed through Origin 2025 software (Origin Lab, Massachusetts, United States).

## 3 Results

### 3.1 Antifungal activities

**Figure 1:**
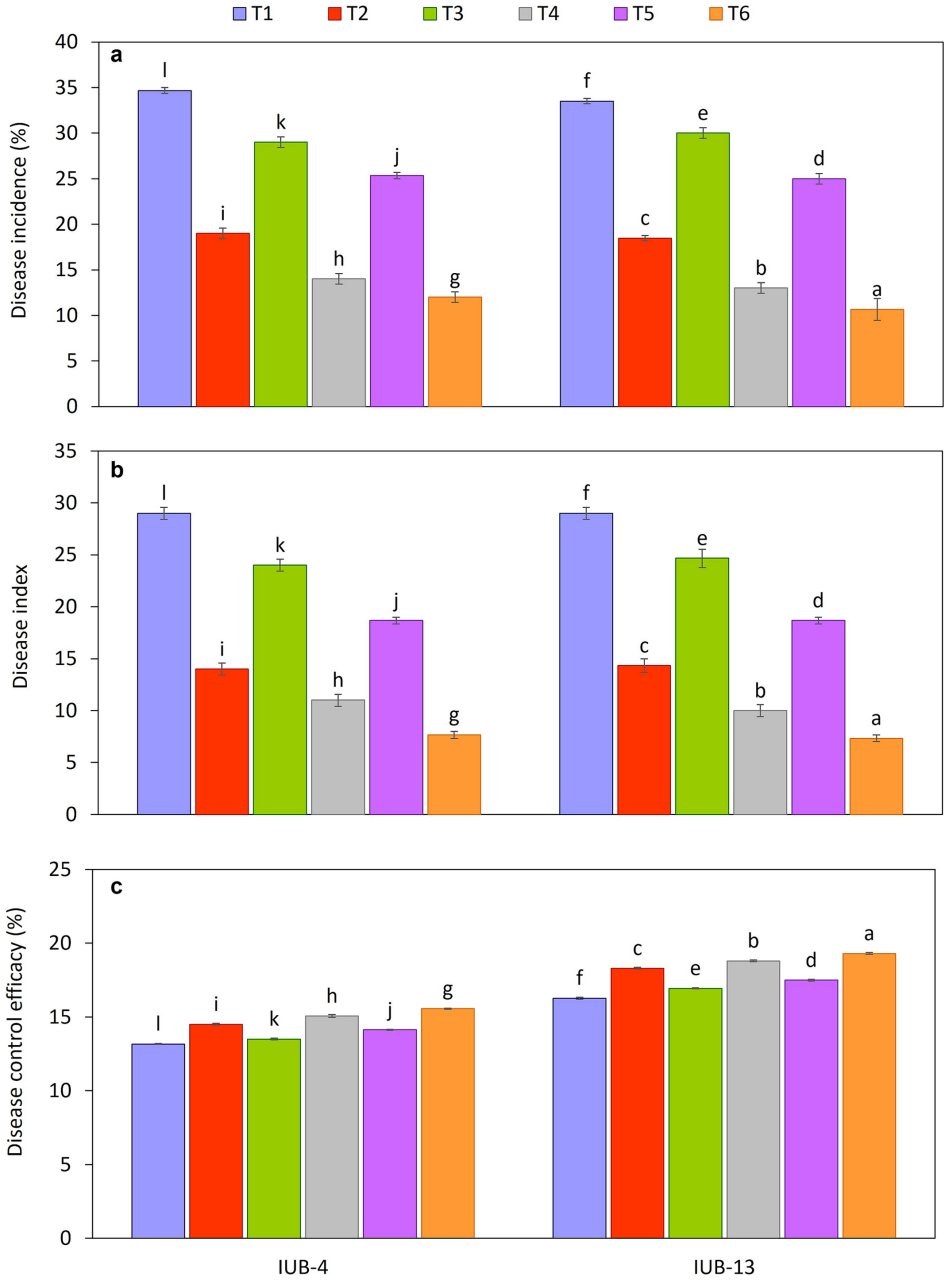
Impact of fertilizer and PGPR-based consortium on antifungal activities of disease incidence **(a)**, disease index **(b)** and disease control efficiency **(c)** of cotton. Bars sharing the same letter (s) are not significantly different from each other at *p* ≤ 0.05.

Consortium spray as a biocontrol agent demonstrated substantial potential in reducing disease incidence and disease index, as illustrated in Figure 1. The effectiveness of the consortium spray was consistent across both varieties tested. The highest disease control (Figure 1a) was observed in the IUB13 and IUB4 varieties, with a maximum reduction of 68 and 65%, respectively, when the consortium spray was used with the recommended NPK + micronutrients + PGPR. Similarly, a reduction of 61 and 60% in disease incidence was noted in the IUB13 and IUB4 varieties, respectively, when the consortium spray was combined with the recommended NPK + PGPR treatment. The disease index also showed a significant reduction under these treatment conditions compared to the application of synthetic chemical sprays (Figure 1b). Specifically, both varieties reduced the disease index by 75 and 74% when the consortium spray was used with the recommended NPK + micronutrients + PGPR treatment. In contrast, the impact of the synthetic chemical sprays on the disease index was not as pronounced as the control in both varieties. Control efficiency was also significantly improved in treatment where the PGPR consortium was sprayed compared to other treatments (Figure 1c). However, the impact was highest in T6, where PGPR was sprayed and recommended NPK + micronutrients + PGPR was applied.

### 3.2 Cotton growth and yield

#### 3.2.1 Root parameters

**Figure 2:**
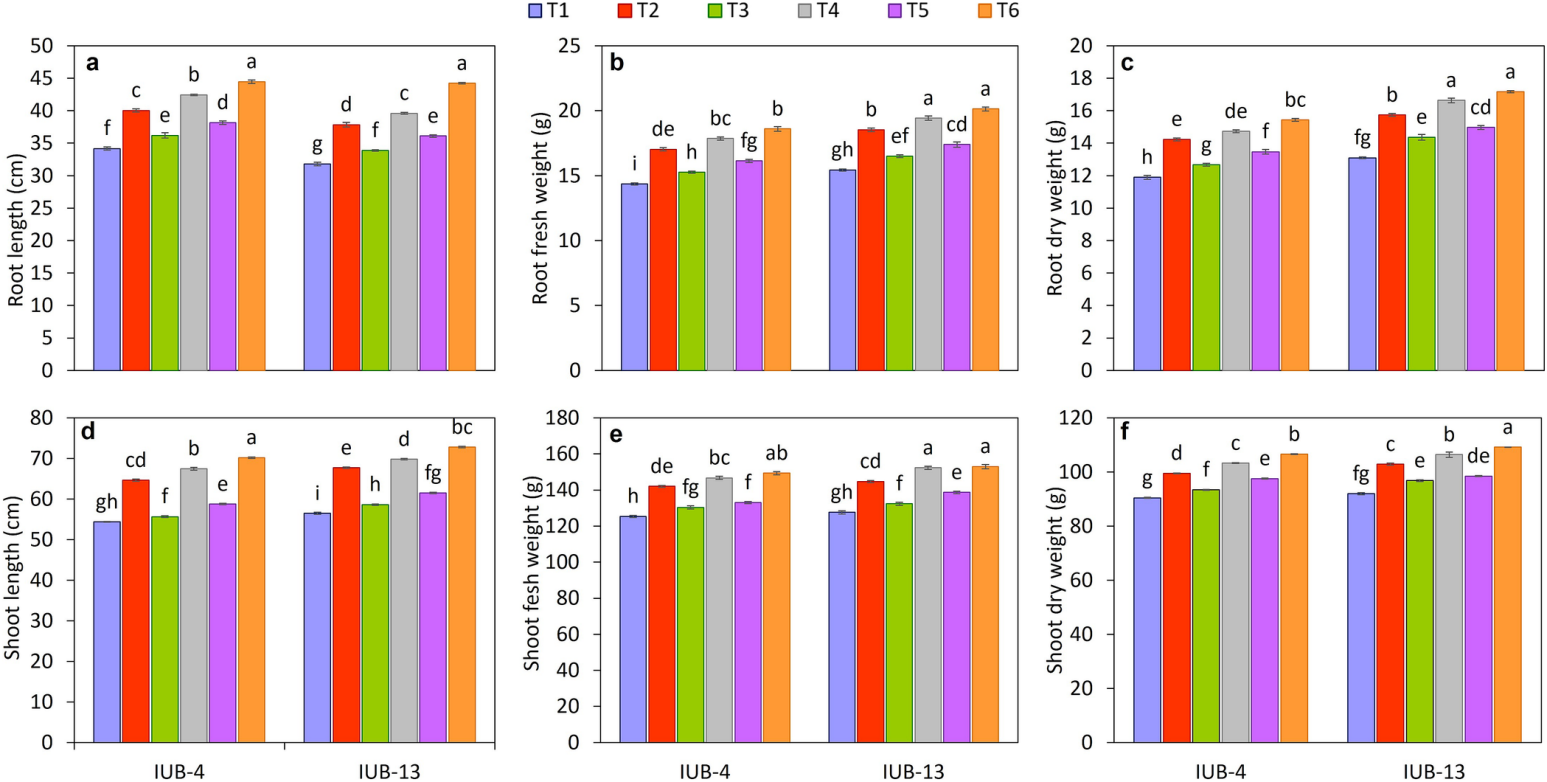
Impact of fertilizer and PGPR-based consortium on cotton growth as root length **(a)**, root fresh weight **(b)**, root dry weight **(c)**, shoot length **(d)**, shoot fresh weight **(e)** and shoot dry weight **(f)**. Bars sharing the same letter (s) are not significantly different from each other at *p* ≤ 0.05.

The PGPR significantly affected root growth parameters of cotton varieties IUB13 and IUB4 when recommended NPK and micronutrients were also applied over control (Figures 2a–c). It was evident from the experiment that NPK + micronutrients + PGPR (T6) increased the root length and fresh and dry biomasses. The highest increase in root length of IUB13 and IUB4 was 39 and 30%, followed by 40 and 24% under recommended NPK + micronutrients + PGPR and recommended NPK + PGPR application, respectively, compared to respective controls. Root fresh and dry biomasses were also significantly increased due to the application of recommended NPK + micronutrients + PGPR, the increase was 30 and 29% in fresh biomass and 31 and 30% in dry biomass under IUB13 and IUB4, respectively. All the treatments were significant compared to the control but non-significant compared to each other. The application of recommended NPK was the treatment that showed the minimum increase in root growth parameters for both cotton varieties.

#### 3.2.2 Shoot parameters

Inoculating PGPR in cotton varieties IUB13 and IUB4 and applying combinations of the recommended NPK and micronutrients improved the shoot length and shoot fresh and dry biomass. Data showed (Figures 2d–f) that the highest increase in shoot length of cotton variety IUB13 was in treatment where recommended NPK + micronutrients + PGPR were used. The increase was observed by 19%, followed by the treatment of recommended NPK + PGPR (15%) compared to the control. Application of recommended NPK + micronutrients + PGPR also performed well for improving the shoot fresh and dry biomass of both cotton varieties, where the increase was 20 and 19% in fresh biomass and 19 and 18% in dry biomass as compared to the control in IUB13 and IUB4, respectively. The statistical data showed that all the treatments had caused significant improvements in the shoot parameters compared to the control treatment. However, these were non-significant as compared to each other.

#### 3.2.3 Yield attributes

Data regarding single boll weight and lint percentage of cotton varieties IUB13 and IUB4 are depicted in Table 2. It was clear from the statistical data that all the treatments performed better in increasing the single boll weight and lint percentage of the cotton crop. Treatment with recommended NPK + micronutrients + PGPR (T6) caused 32 and 29% increases in single boll weight over the controls of IUB13 and IUB4, respectively, followed by recommended NPK + PGPR, which showed 28 and 23% increases in single boll weight, respectively. The recommended NPK (T3) treatment was the non-significant treatment, which showed a minimum increase in single boll weight that was 17 and 5% over the respective controls in the case of IUB13 and IUB4, respectively. The lint percentage was also improved by the treatment of recommended NPK + micronutrients + PGPR, which was 13 and 12% in IUB13 and IUB4, respectively, over the respective controls.

**Table 2:** Impact of fertilizer and PGPR-based consortium on yield of cotton and soil organic matter of rhizosphere soil.

Treatment	Single boll weight (g)		Lint percentage (%)		Organic matter (%)	
	IUB13	IUB4	IUB13	IUB4	IUB13	IUB4
Control	2.17 ± 0.03^fg^	2.07 ± 0.03^g^	30.87 ± 0.19^h^	29.7 ± 0.06^i^	0.58 ± 0.12^fg^	0.57 ± 0.13^g^
PGPR	2.7 ± 0.03^abc^	2.37 ± 0.03^de^	33.7 ± 0.06^c^	32.3 ± 0.06f	0.63 ± 0.37^c^	0.64 ± 0.27^d^
Recommended NPK	2.53 ± 0.03^cd^	2.17 ± 0.03^fg^	32.6 ± 0.06^ef^	30.5 ± 0.06 h	0.59 ± 0.25^e^	0.59 ± 0.21^f^
Recommended NPK + PGPR	2.77 ± 0.03^ab^	2.53 ± 0.03^cd^	34.23 ± 0.07^b^	32.77 ± 0.03e	0.64 ± 0.51^b^	0.64 ± 0.16^c^
Recommended NPK + Micronutrient	2.63 ± 0.03^bc^	2.27 ± 0.03^ef^	33.2 ± 0.06^d^	31.27 ± 0.03 g	0.60 ± 0.14^de^	0.60 ± 0.11^e^
Recommended NPK + Micronutrient + PGPR	2.87 ± 0.03^a^	2.67 ± 0.03^bc^	34.9 ± 0.06^a^	33.4 ± 0.06 cd	0.66 ± 0.18^a^	0.65 ± 0.22^b^
HSD (*p* ≤ 0.05)	0.1708		0.3856		2.5891	

Note: the treatments sharing similar letters didn’t differ significantly at *p* = 0.05 as per tuckey’s test.

### 3.3 Antioxidant enzyme activities

**Figure 3:**
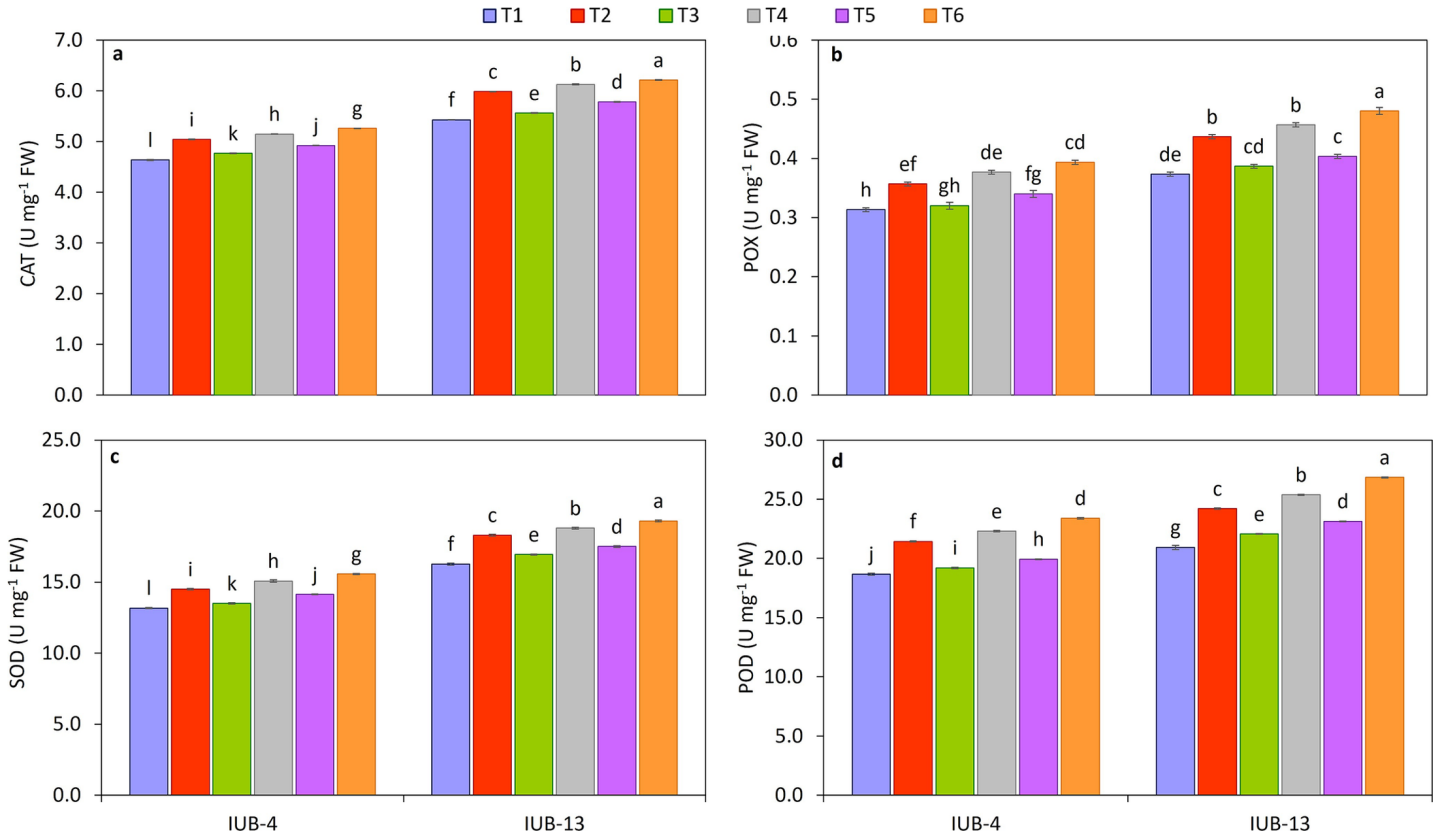
Impact of fertilizer and PGPR-based consortium on CAT **(a)**, POX **(b)**, SOD **(c)**, and POD **(d)** of cotton plants. Bars sharing the same letter (s) are not significantly different from each other at *p* ≤ 0.05.

The antioxidative activity of SOD, POX, POD, and CAT enzymes in cotton varieties IUB13 and IUB4 leaves is presented in Figures 3a–d. A significant increase in enzymatic status was observed with the combined application of recommended NPK + micronutrients + PGPR. Most of the treatments were statistically significant compared to the control except for the single use of recommended NPK. Maximum increase in SOD (19%), POX (29%), POD (28%), and CAT (14%) was observed due to the application of recommended NPK + micronutrients + PGPR, followed by 16, 22, 21, and 13% increase in SOD, POX, POD, and CAT over the control because of the use of recommended NPK + PGPR, respectively. While the maximum increase in the case of IUB4 was SOD (18%), POX (26%), POD (25%), and CAT (13%) over the control due to the treatment of recommended NPK + micronutrients + PGPR. Recommended doses of NPK were the treatment that showed a minimum increment in antioxidative activity in the case of both cotton cultivars IUB13 and IUB4 as compared to the control.

### 3.4 Nutrients uptake

#### 3.4.1 Nutrient concentration in root

**Figure 4:**
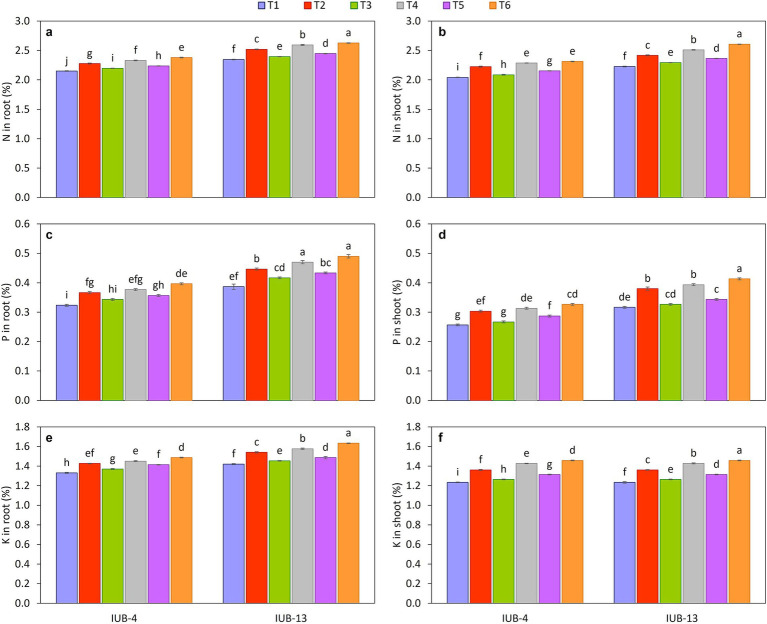
Impact of fertilizer and PGPR-based consortium on Macronutrients concentration in root and shoot, N in root **(a)**, N in shoot **(b)**, P in root **(c)**, P in shoot **(d)**, K in root **(e)**, K in shoot **(f)**. Bars sharing the same letter (s) are not significantly different from each other at *p* ≤ 0.05.

**Figure 5:**
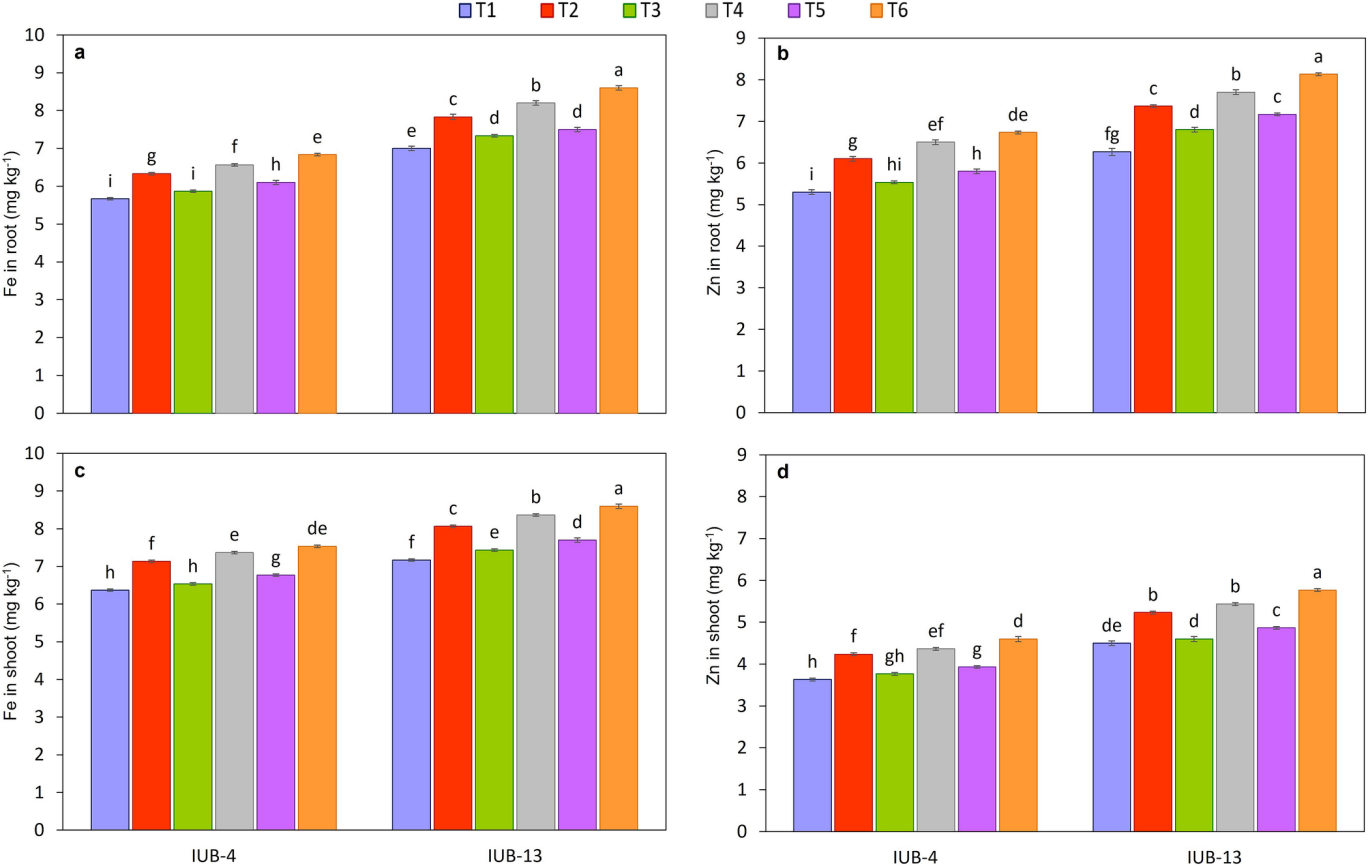
Impact of fertilizer and PGPR-based consortium on Fe **(a)** and Zn **(b)** concentration in roots and Fe **(c)** and Zn **(d)** concentration in roots of cotton plants. Bars sharing the same letter (s) are not significantly different from each other at *p* ≤ 0.05.

The impact of different fertilizer combinations with the PGPR-based consortium considerably improved nutrients in the roots of cotton varieties IUB13 and IUB4. Macronutrient concentration is presented in Figures 4a,c,e, and the micronutrients are shown in Figures 5a,b. The statistical data showed that treatment with recommended NPK + micronutrients + PGPR significantly improved the crop roots’ NPK Fe and Zn concentrations. Maximum increases in NPK contents were 12, 27, and 15% over the respective controls in IUB13. All the treatments were significant except the sole application of recommended NPK. The Fe and Zn concentration increase was 23 and 30% in IUB13 and 21 and 27% in IUB4 due to the treatment of recommended NPK + micronutrients + PGPR. However, applying the recommended NPK showed a minimum increase in nutrient concentration in the roots of both cotton varieties.

#### 3.4.2 Nutrient concentration in shoot

The effectiveness of fertilizer application and PGPR inoculation for improving the macro and micronutrient status in shoots of cotton varieties IUB13 and IUB4 are presented in Figures 4b,d,f, 5c,d, respectively. The minimum increase in nutrient concentration in shoots was noted due to the impact of recommended NPK in both cotton varieties. While the maximum increase was because of the treatment of recommended NPK + micronutrients + PGPR, which increased NPK 17.0, 31.0 and 22.0% in case of IUB13 and 13.0, 27.0 and 18.0% in case of IUB4, respectively, while the increase in Fe and Zn was 20 and 28% in IUB13 and 18 and 27% in IUB4, respectively over their respective controls as showed in Figures 5c,d. Data showed an increase in nutrient concentration in shoots by the recommended NPK treatment, which was non-significant and lowest among all the treatments.

### 3.5 Soil health indices

**Figure 6:**
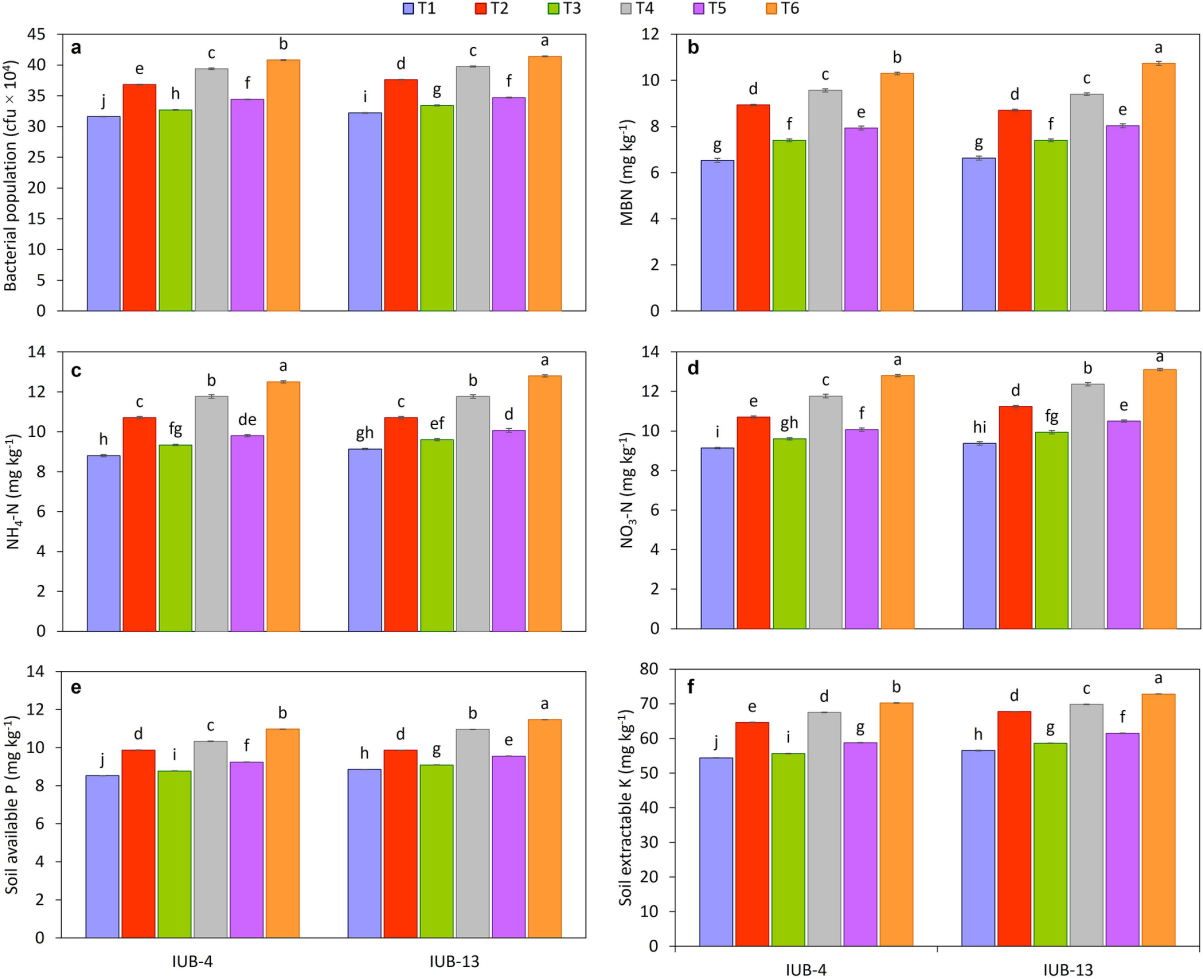
Impact of fertilizer and PGPR-based consortium on biological and chemical properties of rhizospheric soil, bacterial population **(a)**, microbial biomass nitrogen [MBN] **(b)**, NH_4_-N **(c)**, NO_3_-N **(d)**, soil available P **(e)**, soil extractable K **(f)**, of cotton plants, values sharing the same letter (s) are not significantly different from each other at *p* ≤ 0.05.

The results demonstrate a significant improvement in the post-harvest biological properties of the rhizosphere soil in cotton under the treatment of recommended NPK + micronutrients + PGPR (T6) compared to the other treatments (Figure 6). The bacterial population was highest under T6, with values of 41 × 10^4^ cfu in both IUB13 and IUB4 varieties, marking a significant increase over the control, which recorded the lowest bacterial population at 32 × 10^4^ cfu (Figure 6a). Similarly, microbial biomass nitrogen (MBN) showed a considerable increase under T6, with values of 10.73 mg kg^−1^ in IUB13 and 10.3 mg kg^−1^ in IUB4, compared to the control, which exhibited the lowest values of 6.63 and 6.53 mg kg^−1^, respectively (Figure 6b). In terms of organic matter, T6 also showed the highest percentage in rhizosphere soil of both varieties, with 0.66% in IUB13 and 0.65% in IUB4 rhizosphere, which was significantly more significant than the control, where organic matter content was recorded at 0.58 and 0.57%, respectively, (Table 2). Other treatments, such as recommended NPK + PGPR (T4), also demonstrated notable improvements but were less effective than T6. Overall, the integration of PGPR with recommended NPK fertilizers and micronutrients (T6) consistently outperformed all other treatments, significantly enhancing bacterial population, microbial biomass nitrogen, and organic matter in the rhizosphere soil, thereby indicating its potential to improve soil health and fertility post-harvest.

Integrating the PGPR-based consortium with fertilizers significantly improved the post-harvest chemical properties of the soil in both IUB13 and IUB4 cotton varieties. For ammoniacal nitrogen (Figure 6c), the highest mean values were observed by the impact of recommended NPK + micronutrients + PGPR (T6) treatment, recording 12.8 mg kg^−1^ for IUB13 and 12.5 mg kg^−1^ for IUB4, significantly higher than the control, which had the lowest values (9.1 mg kg^−1^ for IUB13 and 8.8 mg kg^−1^ for IUB4). Similarly, nitrate nitrogen levels peaked under the same treatment with 13 and 12.8 mg kg^−1^ for IUB13 and IUB4, respectively (Figure 6d). The available phosphorus content (Figure 6e) also followed this trend, with the recommended NPK + micronutrients + PGPR treatment yielding the highest values (11.47 mg kg^−1^ for IUB13 and 10.98 mg kg^−1^ for IUB4) compared to the lowest values in the control. For extractable potassium (Figure 6f), the same treatment resulted in the maximum extractable potassium levels, which showed a 29% increase in extractable potassium content in both cotton varieties compared to their respective controls. Intermediate increases were observed in other treatments, but they did not match the effectiveness of the fully integrated treatment. These findings highlight the synergistic effect of PGPR and balanced fertilizers in enhancing soil fertility, particularly under nutrient-deficient conditions, demonstrating their potential as a sustainable soil management strategy.

### 3.6 Relationship between observed attributes in response to applied treatments

**Figure 7:**
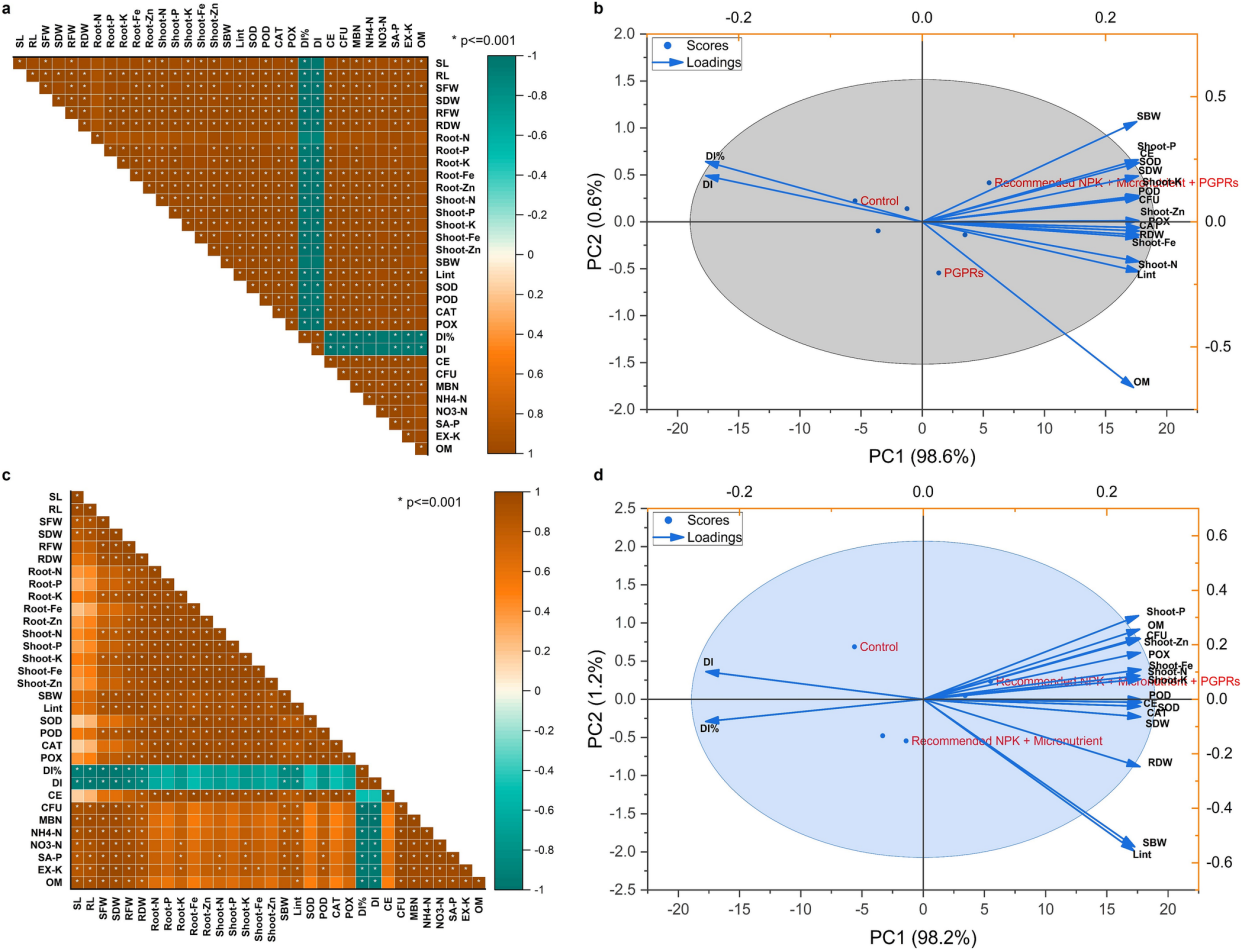
The correlation between the observed attributes in terms of Pearson correlation of IUB4 **(a)** and IUB13 **(c)** and PCA biplot of IUB4 **(b)** and IUB13 **(d)**.

The relationship between the observed attributes of cotton variety IUB-4 and IUB-13 is depicted in Figure 7 in the form of Pearson correlation and PCA biplot. The biplot of PCA depicted that the first and second components of the IUB-13 cotton cultivar showed 98.2 and 1.2% variations in cotton growth, antioxidants status, chlorophyll contents, and yield attributes. On the other hand, the cotton cultivar IUB-4 showed 98.6 and 0.6% variability in growth, antioxidant status, chlorophyll contents, and yield attributes of cotton. Moreover, the negative values of the disease incidence % and disease index for both cotton cultivars depicted suppressing the sooty mold attack by applying the recommended NPK + micronutrient + PGPRs. Pearson’s correlation also described that the growth, yield, and physiological parameters of both cultivars (IUB-13 and IUB-4) were positively correlated by the integration of recommended NPK + micronutrient + PGPRs; however, the disease incidence% and disease index were found to be negatively correlated. These analyses justified our concept of dual action by applying PGPR as a growth promoter of cotton and sooty mold suppressor on the cotton leaf by spraying bacterial consortium at different intervals.

## 4 Discussion

The present study demonstrated the significant impact of PGPR and recommended NPK and micronutrients on cotton growth, yield, nutrient uptake, and disease resistance in the challenging arid climate. Moreover, this investigation highlights the comparison of bacteria and other pesticides for controlling sooty mold. The calcareous sandy soils, characterized by pH (>8) and low organic matter (<0.5%), present substantial hurdles for optimal crop yield. In this context, seed inoculation with PGPR proved to be an effective strategy for enhancing crop productivity by improving nutritional balance and nutrient uptake in crop plants (Fatima et al., 2024; Murad et al., 2024). The consortium of *Bacillus megaterium* (ZR19), *Paenibacillus polymyxa* (IA7), and *Bacillus* sp. (IA16), when applied with recommended NPK and micronutrients, significantly improved cotton growth, yield attributes, nutrient concentrations, antioxidant enzyme activities compared to the control. These strains have previously been reported to enhance crop growth and yield (Ahmad et al., 2021; Iqbal et al., 2022), and our results further confirm their efficacy in cotton cultivation. However, their role in biological control has not been studied earlier. The present investigation filled that gap by determining the incidence of sooty mold attack and resistance caused by the foliar spray of the studied strains compared with chemical pesticides.

The consortium of *Bacillus megaterium* (ZR19), *Paenibacillus polymyxa* (IA7), and *Bacillus* sp. (IA16), when applied with recommended NPK and micronutrients, significantly improved cotton root and shoot growth parameters compared to the control. Another aspect of the study was comparing biological and chemical sooty mold control. The biocontrol potential demonstrated by the PGPR consortium is the key finding of this study. Although sooty mold can significantly reduce cotton yield and quality by interfering with photosynthesis (Belachew and Jenber, 2024), but the result of the present investigation demonstrated a significant reduction in disease incidence and disease index, coupled with high sooty mold control efficacy compared to synthetic pesticides. This reduction of sooty mold under treatments of foliar spray of PGPR may follow the following mechanism, i.e., (i) induction of systemic resistance in cotton against sooty mold (Eski et al., 2019), (ii) reducing honeydew secretions on cotton leaves by killing whitefly and aphids, for example, *Bacillus subtilis* strains is best known biological control agent for cotton whitefly (Çelik et al., 2023; Rumyantsev et al., 2023), (iii) secretions of secondary metabolites with antifungal properties for example, volatile organic compounds (VOCs) inhibiting sooty mold mycelium on leaves (Calcagnile et al., 2022). The strains under study have already proved their potential for VOCs and antibiotics production (Unpublished). Moreover, the improved plant nutrition resulting from PGPR inoculation may enhance disease resistance by inducing systemic resistance. The superior performance of the PGPR consortium compared to synthetic pesticides suggests that these bacteria may offer a more holistic approach to pest management, simultaneously addressing both plant health and pest control. This aligns with the findings by Eski et al. (2019), who reported the remarkable impact of *Bacillus* strains as biocontrol agents for cotton pests. Various *Bacillus thuringiensis* strains have been previously reported as effective biopesticides against different insect orders (Zhang et al., 2009; Eski et al., 2017; Kovendan et al., 2011). The potential of PGPR as an environmentally friendly alternative to chemical pesticides is particularly significant in sustainable agriculture, offering a way to reduce the environmental impact of cotton cultivation while maintaining or improving yields.

The growth enhancement can be attributed to multiple mechanisms adopted by PGPRs, i.e., production of phytohormones like auxins, cytokinins, and gibberellins, which directly stimulate root and shoot growth (Ahmad et al., 2021; Iqbal et al., 2022). Additionally, these bacteria can solubilize phosphates and mobilize other nutrients, making them more available to plants. The production of phytohormones and the nutrient solubilization ability of the PGPR strains might be the possible reason for the observed increases in growth parameters like root length, biomass, and shoot parameters. The synergistic effect of PGPR with NPK and micronutrients suggests that the bacteria may enhance the nutrient use efficiencies of NPK fertilizers and reduce their fixation in the soils of arid climates. These findings align with the findings of Ejaz et al. (2020), who reported that microbially secreted hormones and microbially enhanced nutrient efficiencies are possible reasons for higher cotton production. Chen et al. (2023) also found similar positive effects on tillering, spike length, and grain production in wheat. Moreover, the higher phosphorus solubilization by the applied *Bacillus* strains might be another reason for growth enhancement, as described by Bahadir et al. (2018) and Anwar et al. (2024). Grossi et al. (2024) described another reason for growth enhancement in crops: crop growth enhancement is linked with microbially produced auxin in the rhizosphere, which modifies the root architecture for higher nutrient use efficiencies and water intake. The better development of roots and photosynthesis is responsible for the higher yield of the plants, and our results are in line with the findings of Saleem et al. (2021), who demonstrated the role of IAA-producing rhizobacteria in improving the vegetative growth and yield of cotton.

The improvement in antioxidative enzyme activities in cotton plants treated with the PGPR consortium-recommended NPK and micronutrients is a noteworthy finding that sheds light on the stress tolerance mechanisms of PGPR. The observed increases in SOD, POX, POD, and CAT activities suggest an improved capacity to manage oxidative stress caused by abiotic factors (high temperature of arid climate) and sooty mold attack. This improvement can be attributed to the modulation of gene expression by PGPR under stress. PGPRs potentially upregulate genes involved in antioxidant production in biotic or abiotic stress (Noreen et al., 2024; Ahmad et al., 2023). Our findings are consistent with studies by Li and Jiang (2017), who found that *Bacillus aquimaris* DY-3 significantly enhanced the activities of catalase, superoxide dismutase, peroxidase, and ascorbate peroxidase in maize under salt stress. Similarly, Khan et al. (2020) observed increased SOD activity by 3.1 folds in soybean plants inoculated with PGPR strains SA1 under heat stress. The enhanced CAT activity suggests improved capacity to neutralize hydrogen peroxide, a common reactive oxygen species produced under stress (Fatima et al., 2024; Pandey et al., 2017). These results align with Santos et al. (2018), who reported increased CAT and SOD activities in cowpea nodules co-inoculated with bacteria. This improved antioxidative status likely contributes to the overall enhanced growth and yield observed in the treated plants by mitigating the negative impacts of high temperatures in arid areas.

The results of the present study highlight significant improvements in post-harvest soil biological and chemical properties. The bacterial population in the rhizosphere was notably higher under T6 compared to other treatments, demonstrating the ability of PGPR to enhance microbial activity and diversity. These findings are consistent with the work of Gouda et al. (2018), who reported that PGPR enhances microbial population in the crop rhizosphere due to the secretion of carbohydrates in root exudates and improved soil nutrient availability. Similarly, the increase in microbial biomass nitrogen (MBN) and organic matter observed under T6 aligns with studies by Hussain et al. (2019), which documented that PGPR enhances microbial-mediated nutrient cycling, contributing to higher MBN and organic matter percentage. The enhancement of ammoniacal and nitrate nitrogen and available phosphorus under T6 is consistent with studies by Murad et al. (2024) and Noreen et al. (2024), who demonstrated that PGPR improves mineralization and availability of essential nutrients. This improvement is particularly valuable in calcareous soils (the soil under study), where phosphorus availability is often restricted due to fixation. The observed differences in the response of the two cotton varieties to the treatments may be attributed to their genetic makeup, which influences their nutrient uptake efficiency, antioxidant enzyme activity, and inherent stress tolerance. Additionally, variations in root architecture, nutrient assimilation capacity, and interaction with PGPR strains could contribute to the differential responses. Our findings provide strong evidence for the role of PGPRs in integrated nutrient management and pest management to enhance soil health, crop productivity, and environmental degradation by fertilizers and pesticide application. Future studies should further explore the long-term impacts of these practices on soil fertility and their scalability for broader agricultural applications.

## 5 Conclusion

This study demonstrated the potent efficacy of PGPR combined with recommended NPK fertilizers and micronutrients in enhancing cotton growth, yield, and soil properties. The consortium of *Bacillus megaterium* (ZR19), *Paenibacillus polymyxa* (IA7), and *Bacillus* sp. (IA16) significantly improved antioxidant enzyme activities, growth parameters, yield, and nutrient concentrations in cotton varieties IUB13 and IUB4. Additionally, PGPR integration significantly enhanced post-harvest soil properties, showcasing the synergistic effect of biological and chemical approaches. Integrating PGPR with balanced fertilization is particularly significant for improving cotton productivity and mitigating the impact of sooty mold in arid regions, where nutrient availability and disease pressure are major challenges. These findings underline the role of PGPR in improving nutrient uptake and soil fertility, which is crucial for sustainable cotton production. This integrated approach not only boosts cotton productivity but also reduces dependence on chemical inputs. The PGPR consortium also proved effective in controlling pests, offering an eco-friendly alternative to synthetic pesticides. This dual benefit of growth promotion and pest control is vital for sustainable cotton cultivation. Future adoption of PGPR-based strategies by farmers could enhance yields and soil health, while policymakers can support this transition through field trials and awareness campaigns. Further research on the long-term effects of PGPR on soil health and cotton production is needed to explore its full potential.

## Data Availability

The original contributions presented in the study are included in the article/supplementary material, further inquiries can be directed to the corresponding authors.
